# Significance and Suppression of Redundant IL17 Responses in Acute Allograft Rejection by Bioinformatics Based Drug Repositioning of Fenofibrate

**DOI:** 10.1371/journal.pone.0056657

**Published:** 2013-02-20

**Authors:** Silke Roedder, Naoyuki Kimura, Homare Okamura, Szu-Chuan Hsieh, Yongquan Gong, Minnie M. Sarwal

**Affiliations:** 1 Transplant Research Program Sutter Health Care, California Pacific Medical Center, San Francisco, California, United States of America; 2 Department of Cardiothoracic Surgery, Stanford University School of Medicine, Stanford, California, United States of America; Université Paris Descartes, France

## Abstract

Despite advanced immunosuppression, redundancy in the molecular diversity of acute rejection (AR) often results in incomplete resolution of the injury response. We present a bioinformatics based approach for identification of these redundant molecular pathways in AR and a drug repositioning approach to suppress these using FDA approved drugs currently available for non-transplant indications. Two independent microarray data-sets from human renal allograft biopsies (n = 101) from patients on majorly Th1/IFN-y immune response targeted immunosuppression, with and without AR, were profiled. Using gene-set analysis across 3305 biological pathways, significant enrichment was found for the IL17 pathway in AR in both data-sets. Recent evidence suggests IL17 pathway as an important escape mechanism when Th1/IFN-y mediated responses are suppressed. As current immunosuppressions do not specifically target the IL17 axis, 7200 molecular compounds were interrogated for FDA approved drugs with specific inhibition of this axis. A combined IL17/IFN-y suppressive role was predicted for the antilipidemic drug Fenofibrate. To assess the immunregulatory action of Fenofibrate, we conducted *in-vitro* treatment of anti-CD3/CD28 stimulated human peripheral blood cells (PBMC), and, as predicted, Fenofibrate reduced IL17 and IFN-γ gene expression in stimulated PMBC. *In-vivo* Fenofibrate treatment of an experimental rodent model of cardiac AR reduced infiltration of total leukocytes, reduced expression of IL17/IFN-y and their pathway related genes in allografts and recipients’ spleens, and extended graft survival by 21 days (p<0.007). In conclusion, this study provides important proof of concept that meta-analyses of genomic data and drug databases can provide new insights into the redundancy of the rejection response and presents an economic methodology to reposition FDA approved drugs in organ transplantation.

## Introduction

There is an unmet clinical need for novel immunmodulatory drugs in transplantation, as redundant alloimmune mechanisms, not adequately targeted by current immunosuppressive drugs, require additional modulation to mitigate the development of graft injury, chronic allograft damage and premature graft loss. Better understanding of some of these redundant immune responses may allow for the identification of novel drug targets and drugs for improved post-transplant patient care.

We hypothesized, that the application of a bioinformatics based genomic drug target discovery that uses publicly available functional data in conjunction with the concept of repositioning already FDA approved drugs, represents a promising approach for transplantation medicine which has a finite market size, to identify novel treatment options. This approach has been previously successfully applied by us in inflammatory bowel disease [1], and is now focused on human renal acute allograft rejection (AR).

Initial discovery of escape mechanisms in transplant rejection was done by whole genome microarray analyses of renal transplant recipient biopsies with AR. Analyses focused on bio-databases of functional gene-sets and pathways and discovered biologically relevant transcriptional changes in kidney allograft AR. We identified the Interleukin- (IL) 17 pathway as a pivotal redundant pathway in transplant rejection under the umbrella of Calcineurin inhibitor based immunosuppression (Tacrolimus, Cyclosporine). Recent evidence has hypothesized IL17 as a potential escape mechanism in AR if IFN-y mediated/Th1 responses are suppressed as is with Calcineurin inhibitors [2].

IL17 acts as pro-inflammatory cytokine promoting neutrophil and monocyte recruitment to sites of inflammation usually under the influence of IL-1β, IL-6, and tumor necrosis factor (TNF), and interferon (IFN)-γ [3]. Transcription and production of IL17 during AR occurs in multiple cell-types and is not limited to the Th-subpopulation: IL17 can be expressed by innate and adaptive immune cells, particularly by neutrophils, macrophages, dendritic cells, CD4+ and CD8+ T-cells, in addition to endothelial and epithelial cells [4]–[7]. IL17+ cells in biopsies from kidney transplant recipients correlated with the degree of inflammation during AR and independently predicted graft dysfunction at the last follow up [6]. Our results together with other previously published data suggested that IL17 could be an attractive drug target for transplant medicine [8], [9]. Currently, there is no FDA approved small molecule drug to regulate IL17 responses and antagonizing IL17 in transplantation is not an approved indication.

Bioinformatic analyses of the genomic and drug databases identified Fenofibrate as a drug with established human safety that regulates IL17 and IFN-y responses and thus could be repositioned for treatment of the IL17 mediated axis of allograft AR. Fenofibrate previously attenuated IFN-γ and IL17 mediated experimental colitis [10] and has also reduced systemic inflammatory effects in patients with metabolic syndrome [11] within clinical studies. In addition, we selected Fenofibrate for our drug repositioning studies as it is a small molecule drug for oral application, with proven administration, distribution, metabolism and excretion (ADME) profile, and has extensive human safety data with tolerable side effects.

### Study Design

**Figure 1:**
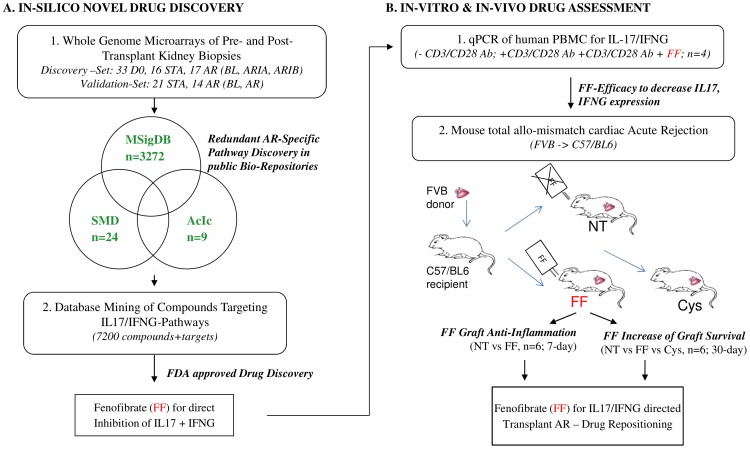
Study Design. Whole genome microarrays from 66 pre- and post-transplant kidney graft biopsies and from 35 post-transplant kidney graft biopsies were analyzed for rejection specific injury pathways using 3 computational databases comprising 3305 independent gene-sets. AR specific IL17 pathway (FDR = 0.3, p = 0.011) and activated IL17^+^ T-helper cells cell (FDR = 0.5; p = 0.008) gene-sets were aligned to a database of 7200 compounds with proven interactions with the input genes (MetaCore™, GeneGo, Thomson Reuters) resulting in the identification of Fenofibrate for drug repositioning in AR. Efficacy of Fenofibrate was tested in vitro using human PBMC and in vivo in mouse total allo-mismatch cardiac rejection for anti-inflammatory and for its potency to prolong graft survival.

The study was based on human tissue-specific acute rejection (AR) injury pathway discovery, their application towards inferred drug targets, and the validation of selected drugs for abrogation of experimental graft rejection by in vitro and in vivo methods (Figure 1). The first phase of this study consisted of a whole genome microarray discovery phase in humans for rejection specific transcriptional analyses of implantation (33 D0) and their paired post-transplant rejecting (17 AR) and non-rejecting (16 STA) biopsies. All 66 biopsies were blindly scored by the same pathologist (Neeraja Kambham, Stanford University, CA) and rejection was graded by the Banff criteria [12]. Three computational databases (MSigDb = molecular Signatures database; SMD = Stanford Microarray database; AcIc = activated immune cells) were analyzed for significant enrichment of pathways and networks (GSA = Gene set analysis) that were redundant in AR. For validation, an additional microarray data-set from 35 renal allograft biopsies (21 STA, 14 AR) from adult recipients post transplantation was interrogated which was downloaded from Gene Expression Omnibus (GEO, GSE9493). The IL17 rejection-specific pathway, chosen as one of the most significant pathway in AR in both data-sets was aligned to a drug database with PubMed proven interactions to the input target genes, resulting in the selection of an FDA approved small molecule drug, Fenofibrate, for possible drug repositioning. Fenofibrate was identified to additionally target the IFN-γ pathway. Initial validation of the effect of Fenofibrate was done to examine its regulation of IL17 and IFN-γ in vitro in human peripheral blood mononuclear cells (PBMC) stimulated with anti CD3/anti CD28. Next, Fenofibrate was tested for its efficacy to improve acute tissue rejection and prolong allograft survival in vivo in an experimental model of heterotopic cardiac AR. End-points examined were reduction of inflammation in the graft, and extension of graft survival compared to non-treatment (NT) and standard immunosuppression (Cyclosporine, Cys) applying Flow cytometry (FACS), histology and assessment of graft function by palpation. Local and systemic efficacy of Fenofibrate to regulate IL17 pathway and IFN-y pathway response genes was characterized by quantitative PCR (QPCR) in mice grafts and spleens at post-operative day (POD) 7 and compared to NT and Cys (Figure 1).

## Materials

### Patients and Transplant Biopsies

In our data-set of 66 renal transplant biopsies from 33 unique pediatric and young adult patients (age range 4–22) were examined. To minimize baseline confounders inclusion criteria were young donor age (7–30 years), no delayed graft function, unsensitized recipients (peak panel reactive antibody <20%), and similar immunosuppression (Daclizumab induction, mycophenolate mofetil (MMF), tacrolimus (Tac)) (Table 1). Excluded were expanded criteria donor kidneys, as they are not used for pediatric and adolescent recipients. Each patient had a biopsy profiled at time of engraftment (D0) prior to reperfusion of the donor kidney, and within the first year post-transplantation, either for cause or as part of protocol surveillance. Biopsies were blindly read for histological evaluation by a single pathologist resulting in 17 patients with Banff graded [12] acute rejection (AR), and 16 patients with essentially normal biopsy reads (no substantive pathology, negative C4d, CD20 staining) (STA). In the rejection cohort, 4 biopsies showed relevant cellular infiltrates that were suspicious for but did not reach the threshold for diagnosis of acute T-cell mediated rejection and were thus diagnosed as borderline rejection (BL), 7 biopsies were graded as ARIA and 6 biopsies as ARIB. All patients gave written informed consent, and the study was approved by the institutional review board of Stanford University and adhered to the Declarations of Istanbul and Helsinki. Written informed consent was obtained and documented on the consent form from the next of kin, caretakers, or guardians on the behalf of the minors/children participants involved in the study and was approved by the ethics committee [13], [14]. Demographics and clinical information are summarized in Table 1. The publicly available data-set (GSE9493) downloaded from Gene Expression Omnibus (GEO) included data from 35 renal allograft biopsies from adult recipients post transplantation. This data-set included 21 STA, and 14 rejection cases. Within the rejection group, 4 cases showed abnormalities which were graded as BL rejection according to Banff [15]. Demographics of these patients can be found here [16].

**Table 1:** Patient Demographics.

Score	D0	STA	BL	ARIA	ARIB
**Number**	33	16	4	7	6
**Histological diagnosis ** ***(Banff)***	Pre-transplant donor biopsy	No significant graft abnormalities	Borderline acute rejection	Mild-moderate cellular acute rejection	Severe cellular acute rejection
**% non-protocol**	/	25%	50%	57%	33.3%
**Time post Transplantation ** ***[months]***	/	25.4 (+71/−25)	10.2 (+8/−5)	12.6 (+19/−7)	5.8 (+8/−3)
**# C4D**	/	0	0	2	1
**# CD20**	/	0	0	0	3
**Serum Creatinine ** ***[mg/mL]***	/	0.5275	1.075	0.924	0.72
**Recipient age**	8.3 (+10/−6.7)	16.6 (+2/−4)	12.3 (+6/−4)	11.8 (+6/−8)	13.4(+5/−10)
**Recipient gender ** ***[%male]***	75%	75%	50%	28.6%	66.7%
**Donor age**	29.5(+23/−15)	31.1 (+16/−17)	26.5 (+12/−26)	26.7 (+17.3/−19)	26.7 (+17/−11)
**Donor gender ** ***[%male]***	40.9%	50%	50%	57.1%	83.3%
**Immunosuppression**	/	MMF+Tac (9)/Sir (1)/Aza (1) +/−SB (5)^b^	MMF+Tac+SB	MMF+Tac (4)/Sir(1)+/−SB (2)	MMF+Tac(2)/Aza(1)+/−SB (3)
HLA MM a	/	1.5	0	0	0

a HLA MM = human leukocyte antigen mismatch;

b Sir = Sirolimus; SB = steroid based; Aza = Azathioprine;

### Mice for Experimental Transplant Rejection

For in vivo experimental heart transplantation, recipient C57BL/6J (H2b) and donor FVB (H2q) mice were used (SM and [8]). All animal experiments were approved by Stanford University Institutional Animal Care and performed in accordance with the Guide for the Care and Use of Laboratory Animals (Ref; National Research Council 1996; Guide for the Care and Use of Laboratory Animals, Washington D.C., National Academy Press). All surgery was performed under sodium pentobarbital anesthesia, and all efforts were made to minimize suffering. In brief, FVB donor hearts were implanted into the abdomen of C57BL/6 WT mice representing a complete MHC-class 1 and -2 mismatch [17], [18]. Animals were divided into treatment (Fenofibrate, FF) and no-Treatment (NT) groups, each consisting of 6 animals in the in the 7-day treatment model and consisting of 6 animals in the NT group, 9 animals in the FF group in the 30-day graft survival study. In the graft survival study, we additionally added a group of 6 animals that were treated with standard immunosuppression, Cyclosporine (Cys). Animal activity, body weight and graft function were assessed daily. The latter was measured by direct abdominal palpation and expressed as graft beating score (BS) using the Stanford cardiac surgery laboratory graft scoring system (0: no contraction; 1: contraction barely palpable; 2: obvious decrease in contraction strength, but still contracting in a coordinated manner, rhythm disturbance; 3: strong, coordinated beat but noticeable decrease in strength or rate, distention/stiffness; or 4: strong contraction of both ventricles, regular rate, no enlargement or stiffness).

## Concise Methods

### Human Tissue Microarray Experiments

Total RNA extraction, quality control, complementary (c)-DNA amplification and microarray hybridization for human renal allograft biopsies was essentially performed as published [19] and described in SM. In brief, total RNA was extracted from biopsies stored in RNAlater (Ambion, Texas, TX) using TRIzol Reagent (Invitrogen, Carlsbad, CA). After quality control, RNA was amplified to cDNA, biotin labeled and hybridized onto Affymetrix GeneChip Human Genome U133 plus 2.0 Arrays.

### Human PBMC Stimulation

Human PBMC for in-vitro drug efficacy assays were isolated from whole blood of 5 healthy individuals (2 female, 3 males, mean age 31+/−14 years) using Ficoll gradient centrifugation (Ficoll-Paque™ PLUS, Amersham Biosciences, Uppsala, Sweden). Isolated PBMCs were pretreated with 100 µMol Fenofibrate (Sigma Aldrich, St. Luis, MO) for 2 hours and then stimulated with anti-human CD3/CD28 antibodies for 65 hours. Thereafter cells were harvested and snap frozen at −80°C until downstream analysis of gene expression. In vitro experiments are described in detail in SM.

### Mouse Treatment

Fenofibrate (F6020; Sigma Aldrich, St. Louis, MO) was dosed at 100 mg/kg body weight/day and administered daily either by i.p. injection in the 7-day inflammatory model, or by oral gavage in the 30-day graft survival study. Treatment started the day prior to transplantation and lasted until the day before sacrifice (SM). Cyclosporine (Cys) was dosed at 20 mg/kg/day and was administered daily by i.p. injection. The dosage of Cyclosporine was based on published literature in experimental cardiac allograft rejection [20], the dosage of Fenofibrate was based on literature of an experimental mouse model of atherosclerosis that showed efficacy but no toxicity during the treatment [21]. A 1∶1 translation of either Cyclosporine or of Fenofibrate dosages used to treat humans were not possible, as the ADME profiles between mice and humans are not comparable.

### Treatment Efficacy Endpoints

#### Murine cardiac allograft survival

To investigate cardiac graft survival in transplanted mice, graft beating scores were determined daily for a maximum of 30 days in Fenofibrate (FF, n = 9) treated mice, and was compared to non-treated animals (NT, n = 6), as well as to standard immunosuppression, Cyclosporine (CSA, n = 6) treated animals. Significance between graft survival between the FF, CSA, and NT groups were assessed by Wilcoxon log rank test in GraphPad Prism 5.04 (GraphPad Software Inc., La Jolla, CA). A p-value <0.05 was considered significant.

#### Murine cardiac allograft inflammation

To study anti-inflammatory effects of Fenofibrate in cardiac AR, animals underwent Fenofibrate treatment for 7 days and the following assays were performed:

Graft histology at POD7 was evaluated by light microscopy of hematoxylin and eosin (H&E) stained formalin fixed and paraffin embedded tissue sections using a Nikon E600 light microscope (Nikon Instruments Inc., Melville, NY) at 10× magnification and Spot V4.6 imaging software (Spot Imaging, Sterling Heights, MI). See SM for details.

Fluorescence Activated Cell Sorting (FACS) of graft infiltrating cells was performed to determine the number of infiltrating cells in recipient cardiac allografts (CD4+, CD8+, B220+, CD11b+, F4/80, Gr1.1) at POD7 and has been described by us [8] and can be found in detail in SM.

#### Human in vitro and murine in vivo QPCR

For quantification of gene expression in in-vitro and in-vivo experiments by quantitative PCR (QPCR), total RNA from human PBMC was isolated using the Pico Pure RNA isolation Kit (Arcturus, Life Technologies, Foster City, CA). Total RNA from mice allograft and spleen tissues was extracted using TRIzol® Reagent (Invitrogen, Life Technologies, Carlsbad, CA) [8]. Each time, 250 ng of total RNA were reverse transcribed using Superscript II (Invitrogen, Life Technologies, Carlsbad, CA). In human PBMC, expression of 7 genes plus 18S as endogenous control was analyzed in duplicates by TaqMan QPCR (ABI HT7900 Instrument, Applied Biosystems, Life Technologies, CA, USA) according to standard protocols. Data was analyzed using SDS 2.3 software (Applied Biosystems, Life Technologies, Foster City, CA). Mouse allograft and recipient spleen gene expression was assessed using the high throughput Fluidigm BioMark instrument (BioMark; Fluidigm, San Francisco, CA) as described in detail in SM. In brief, cDNA was amplified for 14 target genes using Applied Biosystems primers and probes. Preamplified cDNA was loaded into a Dynamic 96.96 chip (Fluidigm) for a 40 cycle QPCR. Expression of 18S served as endogenous control, and data was analyzed in the Biomark RT-PCR analysis software V.2.0. Assay IDs are listed in SM.

### Data Analysis

#### Microarray data analysis

Affymetrix HG U133 plus2.0 gene chip CEL files from 66 pre-transplant donor samples (D0, n = 33) and post-transplant Banff graded renal allograft biopsy samples (STA, n = 16; BL, n = 4; ARIA, n = 7; ARIB, n = 6) were uploaded into dChip 2006 software [22] for processing and normalization. Perfect match only for background correction was performed and arrays were checked for single, array and probe outliers before quantile normalization and computing of model based expression values [23]. Only genes with expression values present on each array were used for analyses. Raw data are stored in gene expression omnibus (GEO) under GSE34437, sample IDs used for microarray analyses are listed in Table S1.

To expand the number of patients and to validate the findings in our data-set an additional microarray data-set (Affymetrix HG U133 plus2.0) was downloaded from GEO (GSE9493) consisting of whole genome expression profiles from 21 STA, 4 BL and 10 AR renal allograft biopsies. Data had been preprocessed and normalized (RMA, Quantile normalization, log2 transformation) as described [16], [24].

#### Computational analyses of databases for rejection pathway analysis

Significance analysis of microarrays (SAM, [25]) for two-class and for quantitative gene-set analysis (GSA) [26] was performed to detect rejection specific gene-sets in patient biopsies with AR. Enrichment in AR compared to STA was identified by two-class GSA. To identify pathways with increasing enrichment from D0>STA>BL>ARIA>ARIAB quantitative GSA was applied. GSA uses maxmean statistics and applies a restandardization of genes and sample permutations to estimate false discovery rate (FDR) which means that a gene-set must be unusual both as compared to gene-sets of the same size sampled at random from the set of genes represented by the gene-set, and as compared to itself, when the outcome labels are permuted [27], [28]. Here, the FDR was calculated in 1000 permutations, and significance level was set at an FDR of 0.5. Correlation between gene expression values and phenotype was based on T-test for 2-class GSA and on regression for quantitative GSA. A total of 3307 publicly available and manually curated gene-sets, containing a total of 839,839 genes ( = 20,930 unique genes) were tested.

From the Molecular Signature Database (MSigDb v2.0, Broad Institute) we downloaded 3272 gene-sets curated using seven online pathway databases (KEGG; BioCarta, BioScience Corp., Reactome, Sigma-Aldrich, Signal transduction knowledge environment and signaling gateway), PubMed publications, and knowledge of domain experts. From the Stanford microarray database (SMD, http://smd.stanford.edu) 24 gene-sets for cellular processes were downloaded, and an additional dataset of Activated Immune Cells (AcicDb) consisting of 9 gene-sets were manually created by us using publicly available microarray gene expression data of in-vitro activated T-helper lymphocytes (Th) (Th1, Th2, IL17+ T-helper cells), antigen presenting cells (dendritic cells (DC), natural killer (NK) cells) and cells of the innate immune system (monocytes, neutrophils). Detailed gene-set descriptions are listed in Table S2.

#### Multivariate analysis of gene expression for IL17 pathway and IL17+ T-helper cells genes

To confirm GSA results, unsupervised Principal Component Analysis (PCA) and hierarchical clustering were performed for the IL17 pathway and Th17 gene-set genes in AR and STA (Partek Genomics Suite V.6.6, Partek Inc. USA). In case of multiple array probe-sets for the same gene, the probe-set with the lowest deviation (standard error of mean) across the AR and STA samples was used for the analyses. In cases where different probe-sets for AR and STA revealed lower deviation, both probe-sets were used. In addition we evaluated if there were any specific transcriptional changes in biopsies that were C4d^+^ AR (n = 3) versus C4d^−^ AR (n = 10).

#### Drug discovery targeting IL17 pathway and IL17+ T-helper cells genes

Affymetrix unique probe-IDs for selected IL17 pathway and Th17 gene-set genes were uploaded into MetaCore™, an interactive platform of biologically relevant data to integrate rejection specific genes with annotated functional data of gene-protein, protein-protein, and protein-compound interactions, together with compound and drug content, and gene disease relationships (MetaCore™; GeneGo, Thomson Reuters, St. Joseph, MI). The Drug Lookup feature in MetaCore™ correlated input genes with deposited data from therapeutic, non-therapeutic, and secondary drug interactions that had an underlying data entry in PubMed. Resulting compounds were then filtered for direct inhibition of target genes, number of genes targeted and their FDA status. In addition, the underlying PubMed entry was carefully checked.

#### QPCR data analysis

Relative gene expression in in-vitro and in-vivo experiments was assessed by the delta delta Ct method and fold changes were calculated to either a human or a mouse universal RNA (Stratagene). Significance was calculated in Excel (Microsoft Office 2007, Microsoft Inc. USA) and GraphPad Prism 5.04 (GraphPad Software Inc., La Jolla, CA) using a 2-sided Student T-test and a p-value of 0.05 as threshold for significance.

## Results

### Discovery of Acute Rejection Specific Redundant IL17 Pathway Enrichment in Human Kidney Transplant Biopsies Across Independent Patient-Sets

Significance Analysis of Microarray Data (SAM) based two-class and quantitative Gene Set Analysis (GSA) [25], [29] across 3307 curated biologically relevant gene-sets and across 66 whole genome microarrays identified significantly increasing enrichment of IL17 pathway genes and genes which had shown significant 2-fold upregulation (p-value <0.05) in T-cells experimentally differentiated into Th17 cells and which were summarized in the “Th17 gene-set” in AR based on false discovery rate (FDR) and p-value (Table 2).

**Table 2:** GSA identifies increasing enrichment of IL-17 gene-sets in human renal allograft acute rejection across independent Patient Data-Sets.

	Analysis	Gene-Sets	p-value	FDR
**Discovery Data-Set (n = 66)**				
	AR vs. STA	IL17-Pathway	0.007	0.3
		Th17 gene set	0	0.2
	D0> STA> BL>ARIA> ARIB	IL17-Pathway	0.011	0.3076
**Verification Data-Set (n = 35)**				
	AR vs. STA	IL17-Pathway	0.011	0.33
		Th17 gene set	0.026	0.39
	STA> BL>AR	IL17-Pathway	0.008	0.5

There were total 140 gene-sets significantly enriched in AR in both Data-Sets (FDR = 0.5), the IL-17 pathway and Th17 gene-sets are listed above. Other significant gene-sets that reached the threshold of FDR = 0.5 included gene-sets associated with innate immune cells (Dendritic Cell, Natural Killer Cell, Granulocyte, Monocyte), and innate immune responses (CTLA4 pathway, Toll-like receptor pathway, NFKB targets, PD1 signaling), with Cytokine Signaling (IL-10, IL-2, IL-5, IL-22BP, IL-12) as well as with gene-sets related to Th-differentiation and activation (CD40 signaling, Costimulation, Th1/Th2, Th-Differentiation). Th1 (FDR = 0.3, p = 0.007) and IL-12 (FDR = 0.3, p = 0.011) had higher FDR and p-values compared to the Th17 and IL-17 gene-sets in our data-set and similar values compared to GSE 9493. Other gene-sets were Graft versus Host Disease, Autoimmune Thyroiditis, and Antigen processing.

Across 13 AR graded ARIA or higher and 16 STA two-class GSA revealed enrichment of the Th17 gene-set with an FDR = 0.2, p = 0, as well as of the IL17 pathway gene-set with FDR = 0.3, p = 0.007. Quantitative GSA (qGSA) across D0, matched STA, and rejection biopsies separated by grade of rejection into borderline (BL), ARIA and ARIB showed that the IL17 pathway gene-set additionally was increasingly enriched with D0<STA<BL<ARIA<ARIB (IL17 pathway gene-set FDR = 0.3 p = 0.011) supporting the relevance of the IL17 pathway in AR. To validate these findings, we investigated an independent microarray data-set from 21 STA, and 14 rejection renal allograft biopsy cases which was publicly available in Gene Expression Omnibus (GEO, GSE9493). Two-class GSA across the STA and biopsy confirmed AR (n = 10) showed significant enrichment of both gene-sets IL17-pathway and Th17 sets in these AR (IL17-pathway, FDR = 0.33, p-value = 0.011; Th17 gene-set, FDR = 0.39, p-value = 0.026). Overall, there were 140 gene-sets common in both data-sets which were significantly enriched in AR (FDR = 0.5). As the publicly downloaded data-set also included 4 cases among their 14 rejection cases that were defined as borderline acute rejection, we added a quantitative GSA analysis which revealed that the IL17 pathway was significantly enriched following STA<BL<AR (FDR = 0.5, p-value 0.008). Due to the small numbers of BL cases in comparison to the numbers of STA and AR cases in this data-set, these analyses might be skewed.

**Figure 2:**
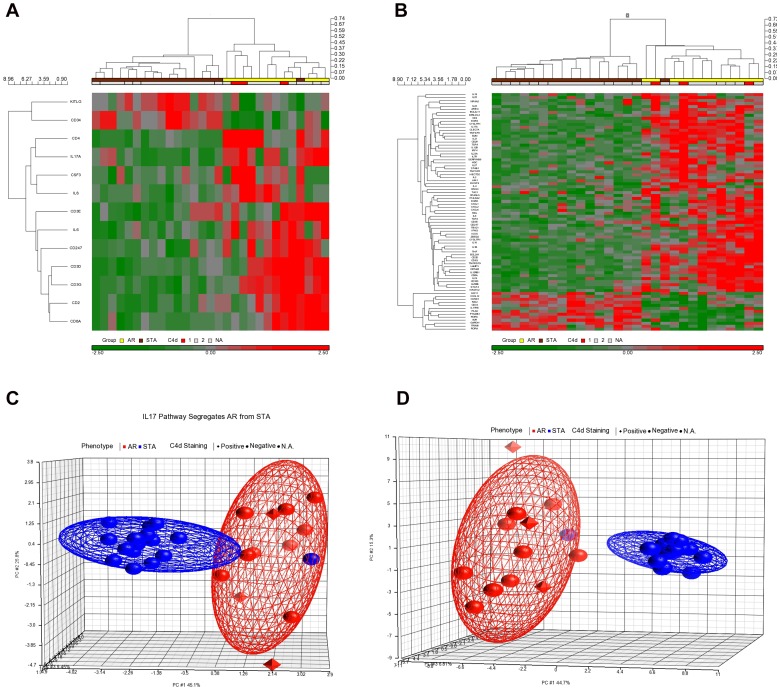
IL17 pathway- and IL17^+^ Th- gene sets in human AR: Segregation of AR and STA. Genes from the IL17 pathway gene-set (2a) and from the Th17 gene-set (2b) were used for hierarchical clustering of post-transplant biopsies and resulted in clear separation of AR and STA after mean centering arrays and genes (Euclidean Distance, Cosine Dissimilarity). Unsupervised principal component analysis (PCA) of the same samples using the same genes confirmed the separation of AR and STA (2c, d). A separation of the AR group into C4d+ and C4d- cases was not seen.

Additional QGSA across 10 gene-sets representing the transcriptome of innate and adaptive immune cells (Monocytes, Natural Killer Cells, Dendritic Cells, Th1-cells, Th2-cells, Th17-cells, T-regulatory cells, gamma delta T-cells) in response to experimental activation, and T-cell differentiation (Th-Differentiation, Anergy/Regulation) identified enrichment of the IL17+ T-helper cell gene-set (Th17, 157 unique genes) [30] in increasing AR (FDR 0.1, p = 0.047; Table 2). The Th17 gene-set response was more redundant in the AR biopsies than the Th1 gene-set response [31] (FDR = 0.1, p = 0.05). Genes regulated in the IL17 pathway and the Th17 gene-sets allowed for almost complete separation of rejecting renal allograft biopsies from non-rejecting biopsies by hierarchical clustering (Euclidean Distance and Cosine Dissimilarity) (Figure 2A, 2B). A total of 8 genes (Il-17 pathway, n = 1; Th17 gene-set, n = 8) were significantly different expressed between C4d^+^ AR vs. C4d^–^ AR cases (Student T-test, p<0.05). Interleukin 8 was common in both gene-sets and showed significant different expression between C4d^+^ and C4d^–^ AR cases. Unsupervised Principal Component Analysis (PCA) for IL17 pathway and Th17 gene-set genes explained 45.1% and 44.7% respectively of the differences between AR and STA in the first of three principal components (Figure 2C, 2D). PCA did not segregate the C4d^+^ AR from C4d^–^ AR.

### Identification of Drugs for Repositioning for IL17 Pathway Directed Inhibition of AR

AR specific IL17 pathway and IL17+ T-helper cell genes were interrogated against the MetaCore™ (GeneGo, St. Joseph, MI) Compound Database evaluating the interaction of rejection specific genes against data of 7200 different therapeutic and non-therapeutic compounds in the drug lookup interface. The resulting 297 therapeutic and non-therapeutic compounds identified to interact with one or more of the input genes from the IL17 and IL17+ T-helper cell gene sets were filtered for direct inhibition and targeting at least 5 genes in the rejection input data-set, including IL17. Fenofibrate, an FDA approved small molecule drug for the treatment of hypertriglyceridemia and hypercholesterolemia, was inferred to directly inhibit IL17A the major IL17 pathway cytokine and was also suggested to act via the Th1response directly inhibiting expression of IFN-γ the major Th1 inducing cytokine [10]. In addition to Fenofibrate, our approach also identified three corticosteroids (Prednisolone, Budesonid and Flunisolide). Steroids are currently used as standard immunosuppression and are the mainstay treatment for an AR episode. This result provided confidence for the validity of our approach. However, the steroids were not predicted to directly act via IL17A. As Fenofibrate was hypothesized to directly act on IL17A as well as on the Th1 cytokine IFN-γ, was an FDA approved small molecule drug with known human safety and side-effect profiles and established drug delivery and administration frequency, and with known pleiotropic anti-inflammatory effects [11], Fenofibrate was selected as drug candidate for AR repositioning. The subsequent in vitro and in vivo experiments in our study support likely efficacy for AR in human renal transplant rejection.

### Attenuation of IL17 and IFN-γ Gene Expression by Fenofibrate in CD3/CD28 Stimulated Human PBMC

**Figure 3:**
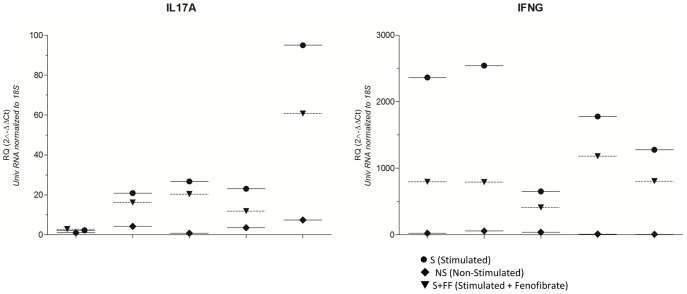
Fenofibrate gene expression in human PBMC: Fenofibrate regulates IL17 and IFN-γ gene expression in CD3/CD28 stimulated human PBMC. PBMC from healthy individuals (n = 5) were stimulated with CD3/CD28 antibodies (S) leading to significant upregulation of IL17 and IFN-γ which was inhibited by Fenofibrate (S+FF). Values represent mean fold changes versus non-stimulated (NS) cells plus Standard error of mean calculated using ΔΔCt method and 18S as endogenous control gene; experiments were performed in triplicates. Student T-test for paired data: * p-value <0.05. Individual p-values are displayed in Table 3.

Anti-CD3/CD28 stimulation of human PBMC from independent healthy volunteers upregulated expression of IL17 and IFN-γ in all five individual PBMC; the average fold change for IL17 was 12.15 (p = 0.01), of IFN-γ 96.58 (p<0.0001) compared to non-stimulated PBMC (n = 5). Fenofibrate treatment of stimulated PBMC significantly reversed the upregulation in all cases but one with respect to IL17 and in all cases with respect to IFN- γ: the average fold change for down regulation was 1.38 and 2.17 for IL17 (p = 0.02) and IFN-γ (p = 0.001) respectively (Figure 3). Shown are relative fold changes (RQ) in gene expression levels normalized to 18S and universal RNA for non-stimulated (NS), stimulated (S) and stimulated and Fenofibrate treated (S+FF) PBMC.

**Table 3:** Effects of Fenofibrate on IFNG and IL17A expression in human PBMC: Fold changes (Fc) comparing stimulated (S) to no-stimulated (NS) PBMC (upper part) and comparing stimulated (S) to stimulated and Fenofibrate treated (S+FF) PBMC (lower part); direction of the gene expression change and p-values calculated by two-sided Student T-test.

		Fc	Direction	P-value
**S vs. NS**	**IFNG**	96.58	Up	*** <0.0001
	**IL17A**	12.15	Up	* 0.0142
**S vs. S+FF**	**IFNG**	2.17	Down	** 0.0014
	**IL17A**	1.38	Down	* 0.0224

### Experimental in-vivo Evaluation of Fenofibrate for Efficacy in AR and Characterization of its Immune Regulatory Effects

#### Prolonged graft survival in a rodent heart transplant model of AR by Fenofibrate treatment

**Figure 4:**
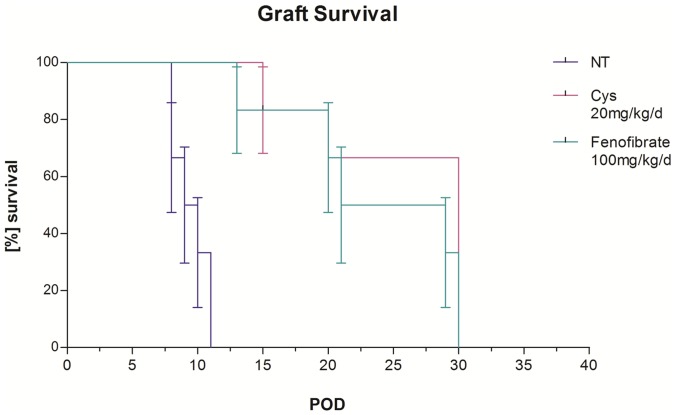
Graft Survival with Fenofibrate: Fenofibrate treatment alone prolonged cardiac graft survival in transplanted mice. Kaplan Meyer curve for graft survival data after total allo-mismatch murine heart transplantation was assessed by determining the number of post operational days (POD) on which transplanted mice showed a palpatable graft beating score (BS). The median number of days grafts of Fenofibrate treated animals (FF, n = 6) showed beating was 25 compared to 9.5 days in non-treated animals (NT, n = 6). Significance of graft survival was assessed by Wilcoxon log-rank test (p = 0.0007).

A 30-day graft survival study in BL6/C57 mice heterotropically transplanted with an allogenic FVB donor heart resulted in significantly prolonged survival of total allo-mismatch donor hearts transplanted into Fenofibrate-treated (100 mg/kg/d) recipients (median survival: 25 days; n = 6) compared to non-treated recipients (NT, median survival: 9.5 days; n = 6) (Wilcoxon log-rank test, p = 0.0007) (Figure 4) and led to allograft beating beyond day 30 (study end-point) in two animals. Mice tolerated the Fenofibrate dose well; no change in total body weight during the treatment was observed. Allografts in the NT control group were rejected within an average of 12 days after transplantation. Comparison to standard immunosuppression (Cyclosporine, Cys, 20 mg/kg/d i.p.) revealed equivalent efficacy of Fenofibrate for extending graft survival (median survival Cys 30 days) (Figure 4).

#### Significantly improved graft function with Fenofibrate

**Figure 5:**
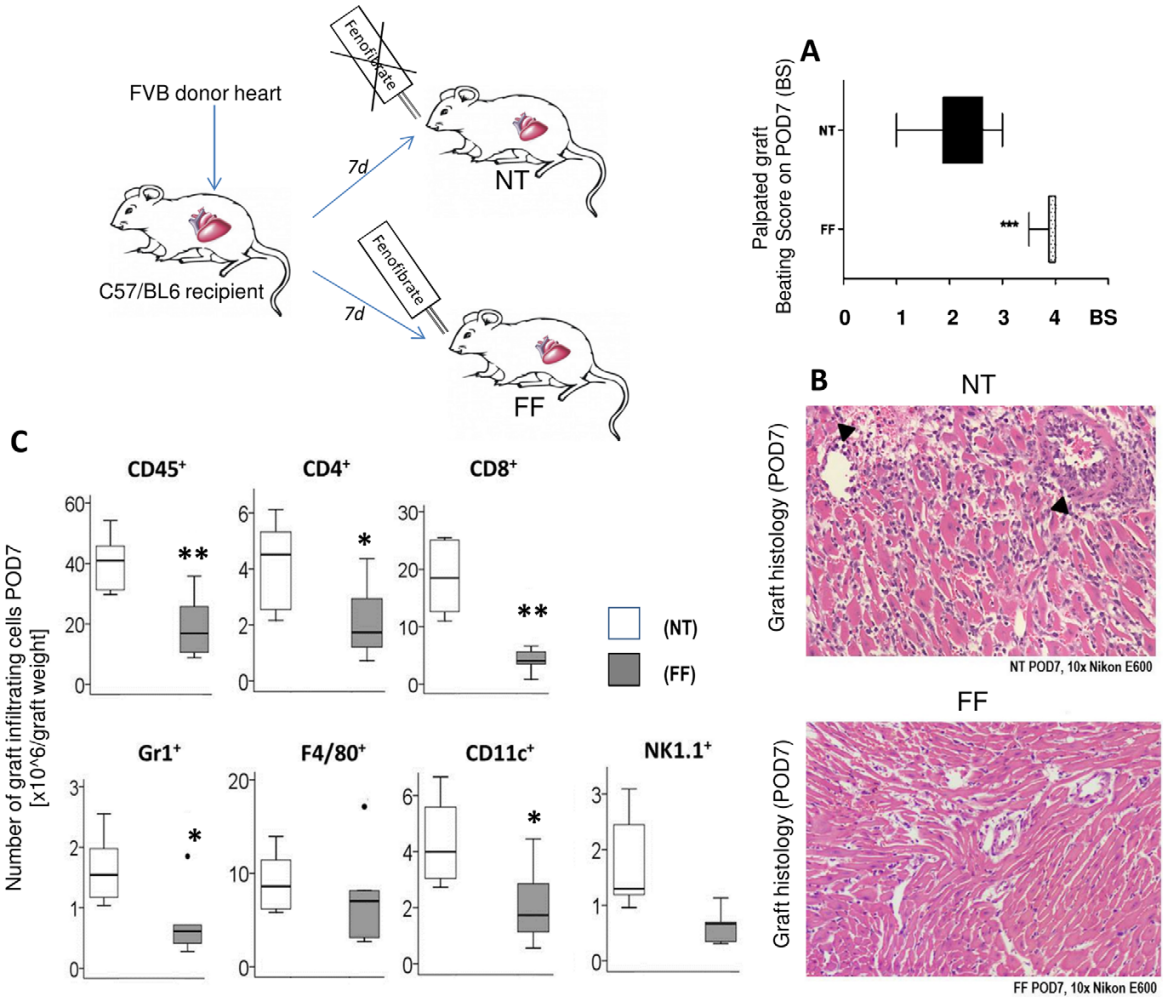
Significant improvement of graft function, graft histology and significant reduction in graft cell infiltration with Fenofibrate treatment. Palpated graft beating in transplanted mice was assessed daily and assigned a beating score (BS). Transplanted mice BS were significantly improved when treated with Fenofibrate (FF) compared to non- treated ones (NT) (n = 6, p<0.001; shown are mean BS plus SEM), (a). H&E stainings of mice grafts at POD7 revealed decreased cellular infiltrates and histological damage upon Fenofibrate treatment (b). FACS of infiltrating cells from the same grafts showed significantly reduced numbers of infiltrating innate and adaptive immune cells in the Fenofibrate treated mice (*p<0.05, **p<0.01, 2-sided Student T-test, n = 6). Box Plots show median and first and third quartiles of infiltrating cells corrected for total number of infiltrating cells and total graft weight.

Seven days daily treatment with Fenofibrate- using the same acute rejection model used for graft survival, resulted in significantly higher average BS’ (mean BS = 3.5) compared to NT (mean BS = 2, p<0.001) (Figure 5A). The chosen dosage of 100 mg/kg/d Fenofibrate was well tolerated by the mice as there was no change in body weight, liver function and serum Creatinine (Materials and Methods S1).

#### Reduction in cellular infiltrates and myocyte damage with Fenofibrate

Graft tissue from POD7 was assessed by a single blinded pathologist by microscopic evaluation of H&E staining applying ISHLT criteria [32], [33]. Grafts from Fenofibrate treated animals revealed decreased cellular infiltrates either uni- or multifocal but never diffuse, and with- or without myocyte damage compared to grafts from untreated animals which always showed diffuse multifocal cellular infiltrates and were always associated with myocyte damage (Figure 5B).

#### Significantly reduced numbers of graft infiltrating innate and adaptive immune cells with Fenofibrate

FACS analysis of graft infiltrating cells not only confirmed histological evaluation but further quantified the anti-inflammatory effect of Fenofibrate. Assessed were total numbers of graft infiltrating total leukocytes (CD45+), and of differential innate Dendritic- (DC,CD11c), Natural Killer (ND, NK1.1), Macrophages (F4/80), Neutrophils (Gr1+)) and adaptive immune Cytotoxic-(CD8), Helper T-(CD4), and B-(CD220)) cells in Fenofibrate and no-treated grafts at POD7 (Figure 5C). Numbers of cells were corrected for total graft weight. Fenofibrate significantly decreased the number of CD45+ graft infiltrating total leukocytes (p<0.001); more specifically, there was a significant reduction in antigen presenting DC (p<0.01), in CD4+ T-helper and CD8+ cytotoxic T-cells, as well as in infiltrating neutrophils (p<0.01) and NK cells. As IL17 is known to promote neutrophil infiltration, the observed reduction of neutrophils in the graft of Fenofibrate treated mice potentially reflects the IL17 pathway inhibition by Fenofibrate. Infiltrating macrophages and B-cells were also reduced but numbers did not reach significance.

#### Significant inhibition of graft and spleen IL17and Th1 genes in vivo with Fenofibrate

**Figure 6:**
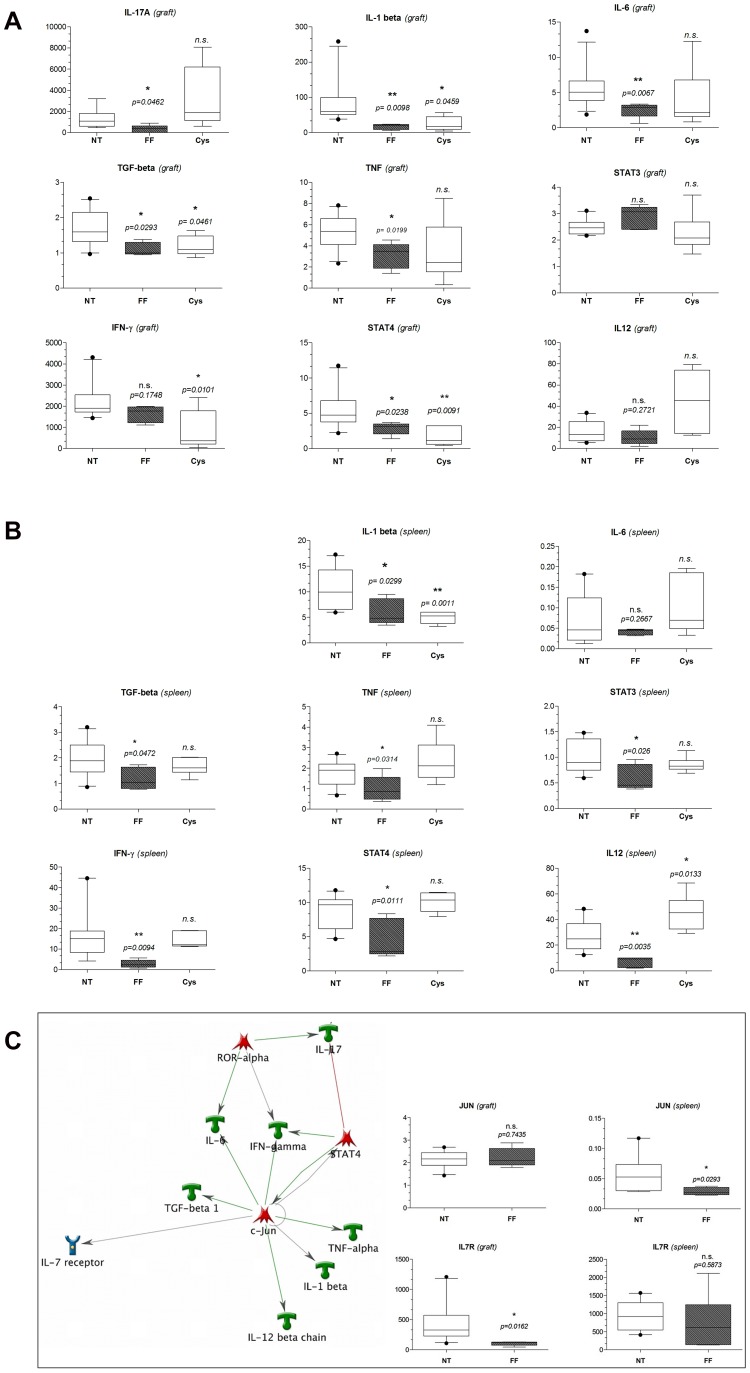
Gene Expression Profiles of Fenofibrate Effects in mouse grafts and spleens: significant repression of IL17 and Th1 genes in vivo in spleens and grafts and formation of a single network of direct interactions. PCR of RNA from mice cardiac allografts (5a) and recipient spleens (b) corroborated in vitro findings and further characterized the mechanism of Fenofibrate to regulate the IL17 pathway and Th1 response (c). Gene expression results in mice recipient grafts (a,c) and spleens (b,c) at POD7 are displayed as box and whisker plots of mean relative fold changes with 10^th^ and 90^th^ percentile to universal RNA after 18S normalization using the ΔΔCt method. Significance between Fenofibrate (FF) treatment over no treatment (NT) and between Cyclosporine (Cys) treatment over no treatment were calculated by a 2-sided Student t-test and a p-value of p<0.05 considered as significant. Results of our additional network analyses in MetaCore revealed a central role for the transcription factor c-jun (c). Subsequent PCR for c-jun and its associated cytokine receptor IL7R showed decreased expression by Fenofibrate and suggested different sites of actions in spleen and grafts (c).

Corroborating the findings of Fenofibrate on anti-CD3/CD28 stimulated human PBMCs, Fenofibrate treatment in vivo in cardiac transplanted mice resulted in reduced gene expression levels of IFN- γ and IL17A (Figure 6A, 6B). IL17A expression in the Fenofibrate treated mice was only detectable in the transplanted hearts and did not reach the threshold of detection by QPCR in the recipients’ spleens at POD7. In grafts IL17 was significantly lower compared to no treatment (6a, p = 0.0462). In spleens, Fenofibrate significantly reduced IFN-γ expression (6b, p = 0.0094) whereas reduction of graft IFN-y expression did not quite reach the significance threshold of p = 0.05 when compared to no-treatment by two sided Student T-test (6a). Next, we further elucidated the efficacy of Fenofibrate and expanded graft (Figure 6A) and spleen (Figure 6B) gene expression analyses to genes up- and downstream of the Th1 response and IL17 pathway: Fenofibrate reduced the IL17 stimulating cytokines IL-1β, and TGF-β, as well as of IL-17 downstream TNF in both mice allografts and spleens. IL-6 which also is upstream of IL17 and together with IL1-β and TGF-β important for activating the IL17 response was significantly reduced by Fenofibrate in mice allografts only. The Th1 response was further investigated quantifying the stimulating IL-12 cytokine expression as well as the Th1 transcription factor STAT4. Fenofibrate significantly decreased expression of both genes in mouse spleens, STAT4 was additionally significantly decreased in the in the graft as compared to mice that were not-treated with Fenofibrate for AR. QPCR in grafts and spleens from Cys treated mice at POD7 validated the preferential inhibition of Th1 responses by Cys seen in reduced graft IFN-y (p = 0.0101) and STAT4 (p = 0.0091) expression, whereas IL17A was not affected by Cys (p = 0.2180) but even showed a trend towards higher expression. To address the question, whether the effect of Fenofibrate to inhibit the IL17 pathway is more dependent on the related orphan receptors RORγ/RORα or on STAT3 (which induce Th17 cell differentiation), we performed additional QPCR for RORγ, RORα and STAT3 expression in grafts and spleens from untreated and Fenofibrate treated mice at POD7. There was no significant difference with treatment in expression of either RORγ (p = 0.26 in the graft; p = 0.59 in the spleen) or RORα (p = 0.96 in the graft; p = 0.076 in the spleen), but there was a significant reduction for STAT3 expression in the spleen of Fenofibrate treated mice at POD7 (p = 0.026), but not in the graft (p = 0.72). Our results indicate that there is bias towards STAT3 targeted IL17 pathway inhibition by Fenofibrate. Additionally, there may be differential immune responses in spleen and graft.

#### Single network formation of rejection specific Fenofibrate regulated genes

Rejection specific genes which were upregulated in AR and down regulated again by Fenofibrate could be assembled into a single-network of direct interactions in MetaCore™ network analyses. In this analysis, c-Jun and downstream of c-Jun, IL-7 receptor (IL7R) were detected as additional genes interacting with the genes that were regulated by Fenofibrate (Figure 6C). Our subsequent analyses of c-Jun and IL7R by QPCR of mice graft and spleen RNA showed that both genes were upregulated in mice which did not receive Fenofibrate and that both were down regulated again in mice treated with Fenofibrate. Specifically, Fenofibrate significantly regulated c-Jun (p = 0.0293) in mice spleens, and IL7R (p = 0.0162) in mice allografts. Interestingly, Fenofibrate did not show efficacy on ROR-α, a transcription factor relevant for IL17 expression in IL17+ T-helper cells (Figure 6C).

## Discussion

The present study elucidates a process of microarray-based and bioinformatics-driven discovery of transplant rejection specific functional gene-sets and pathways that are highly relevant to redundant acute renal allograft rejection, in combination with a process by which FDA approved drugs can be tested for repositioning for immunomodulation in human organ transplantation. While most microarray data analysis methods focus on the identification of individual differentially expressed genes in two or more conditions, we focused here on sets of biologically relevant genes in our initial discovery applying gene set analysis (GSA) as a more robust method with reduced false positive results and more likely to reveal redundant biologically relevant pathways underlying graft rejection and describing potential novel drug targets [26], [30], [33]. Next, we investigated drugs that already passed the costly clinical phases of drug development and drug safety in humans for indications unrelated to organ transplantation, but could inhibit identified critical gene-sets and pathways in the allo-immune response. We hypothesized that these target drugs could be repositioned for suppressing the acute allo-immune response, providing a more cost effective means of introducing novel drugs for treatment of acute rejection in organ transplant recipients.

The role of IL17 in innate and adaptive auto- and allo-immune responses has been investigated by several groups, but is still not fully understood. Clearly, IL17 was increased in human acute lung, liver and kidney rejection [4], [34], [35] and promoted early graft inflammation [36]. Results in experimental mouse models of cardiac AR reported that in Th1 transcription factor T-bet deficient mice, an IL17 response was mounted leading to acute graft rejection [2], [5]. In another study IL17 was also involved in the acceleration of AR in a T-bet positive background with a full Th1 response [8]. The role of T-bet for IL17 mediated acute rejection remains controversial, yet most recent evidence suggests, that IL17 plays an accelerated role in a Th1 response suppressed environment [2]. Our results for IL17 expression in grafts from transplanted mice treated with Cys which majorly affects the Th1 response showed higher IL17 expression compared to not-treatment, whereas IFN-y was significantly lower. As current immunosuppressive drugs used in transplantation majorly act via the Th1 response, a potential increased emergence of IL17 in redundant acute rejection seems to be likely, and our microarray analyses provides further evidence for the role of IL17 pathway as an important mechanism of escape in more aggressive acute allograft rejection in patients on standard immunosuppression where IL17 appears to drive the intensity of allograft inflammation. Thus synergistic inhibition of Th1 and IL17 pathways could be very promising and has been suggested by others [5]. On the contrary, Huh et al. found that cardiac glycosides inhibited differentiation of Th17 cells in vitro with high specificity by binding to the transcription factor RORyt [37]. This did not only result in a decreased IL17 transcription and production, but the isolate inhibition of RORyt by cardiac glycosides additionally resulted in reciprocal increased T-cell IFN-y and FOXP3 expression. The results by Huh et al further support our findings that a synergistic inhibition of both IFN-y and IL17 pathways as seen with Fenofibrate may be especially relevant in diseases where both immune axes play a significant role such as in acute allograft rejection.

Despite this evidence, IL17 inhibitors are not currently used in transplantation and in the absence of any available synthetic IL17 inhibitors, we thus pursued the approach of drug repositioning Fenofibrate, a commercially available FDA approved drug, used for treatment of hyperlipidemia, and inferred from our study, to simultaneously inhibit the IL17 pathway and the Th1 mediated IFN-γ pathway in acute graft rejection. Although Fenofibrate has never been used in transplantation, several studies including the FIELD study [11], indicated general anti-inflammatory pleiotropic effects in patients who were treated with Fenofibrate. In addition to Fenofibrate, steroids currently used in the post-transplant management of AR, were noted to also regulate many of the input AR genes in our dataset, supporting the reliability of our approach, though it is important to note that steroids did not regulate IL17. As Fenofibrate had been shown to inhibit expression of both the IL17 and the Th1 response gene IFN-γ [10], Fenofibrate represented a very promising candidate for repositioning in transplantation. Thus, we characterized the anti-inflammatory effects of Fenofibrate in organ transplant rejection related to the inhibition of the IL17 and IFN-γ/Th1 responses, both locally in the allograft and systemically in the spleen of rejecting animals. We could show profound attenuation of graft rejection and most importantly Fenofibrate extended graft survival by 11 days over no-treatment and was almost as efficient as standard immunosuppression.

The exact mechanism by which Fenofibrate inhibited the IL17 and the Th1 response in our model is not clear, but our gene expression analyses both in PBMC and in mouse grafts and spleens suggested an effect upstream of IL17, as the IL17 pathway activating genes IL1-β, TGF-β and IL6 were significantly decreased by Fenofibrate. IL6 is known to induce the IL17 pathway promoting the differentiation of IL17 producing T-cells [38], and was also increased in human AR [35], [39]. Importantly inhibition of the Th1 response in IL-6 deficient mice had a synergistic effect on attenuating AR [39], similarly to inhibition of IL-6 in T-bet deficient mice [2], both also leading to decreased IL17. Here, we showed that Fenofibrate treatment of experimental AR resulted in simultaneous inhibition of Th1 response genes and of IL6. Our results further suggest that Fenofibrate also regulates the IL17 pathway independent from the IL17+ T-helper cell specific transcription factors ROR-α and –γ, as these were not significantly reduced in mice treated with Fenofibrate. On the other hand, there was significant down regulation of STAT3 in spleens from mice treated with Fenofibrate. Only very recently, an experimental study of murine in-vitro Th-cell differentiation showed that Fenofibrate inhibited a differentiation of IL17 producing CD4+ T-cells, providing another line of evidence for Fenofibrate on the IL17-pathway [40]. In this study, the authors were able to show a dose response curve on IL17 producing T-cells with Fenofibrate [40]. They used TGF-β, IL6, IL4 and IFN-γ to induce the IL17 producing CD4+ T-cells, all of which were upregulated in our study of experimental AR, and again down regulated with Fenofibrate.

We also provided additional data on the mechanism of Fenofibrate by our network analyses, where c-Jun was found to actually act on all genes regulated by Fenofibrate, either directly or indirectly. The protein c-Jun in combination with c-Fos forms the activator protein 1 (AP-1) early response transcription factor. Inhibition of AP-1 using decoy oligonucleotides at day of transplantation was efficient in attenuating cardiac vasculopathy in rats [41]. Here, c-jun was significantly upregulated in mice allografts with AR, and Fenofibrate treatment led to significant down regulation of c-Jun in cardiac grafts compared to no treatment, suggesting a potential central anti-inflammatory mechanism of Fenofibrate on T-cells.

A retrospective analysis of human transplant recipients receiving Fenofibrate is currently under way to evaluate any synergistic protective role of Fenofibrate on acute and chronic graft rejection (Roedder et al, manuscript under preparation). Additional analysis of the genome wide association study of inflammatory biomarker changes in response to Fenofibrate treatment [42] can shed additional light on selecting patients prior to transplantation that can benefit from a combination of Fenofibrate treatment with standard immunosuppression. This data supports that, in addition to standard immunosuppression, the inhibition of the IL17 pathway, may be effective in reducing the incidence and severity of acute rejection and thus positively impact on long term outcomes after organ transplantation and can potentially be achieved using Fenofibrate.

## Supporting Information

Table S1 **Samples used for Microarray Analysis.**
(DOCX)

Table S2 **Gene-sets of innate and adaptive immune cells (AcIc).**
(DOCX)

Materials and Methods S1 **Supporting materials and methods.**
(DOCX)
