# Supplementary material for: Significance and Suppression of Redundant IL17 Responses in Acute Allograft Rejection by Bioinformatics Based Drug Repositioning of Fenofibrate
Source: PLoS One. 2013 Feb 20;8(2):e56657. doi: 10.1371/journal.pone.0056657 (PMC3577752; doi:10.1371/journal.pone.0056657)
Supplement: Table S1 — Samples used for Microarray Analysis. (DOCX) [file pone.0056657.s001.docx]

**Table S1:** Samples used for Microarray Analysis

| **Array Name** | **Group** | **Primary Diagnosis** |
| --- | --- | --- |
| KT979_01-30-07 | ARIA | "AR IA, recurrent GN" |
| KT496_01-30-07 | ARIA | ARIA |
| KT1043_02-12-07 | ARIA | ARIA |
| KT1100_02-14-07 | ARIA | ARIA |
| KT863_03-21-07 | ARIA | AR IA+DT |
| KT775_02-22-07 | ARIA | ARIA; suspicious for ARIIA; tubular atrophy |
| KT1086_02-12-07 | ARIA | AR Banff IA + CAN ; positive C4D suggests humoral component; |
| KT1045_02-12-07 | ARIB | ARIB |
| KT942_02-14-07 | ARIB | AR IB |
| KT725_02-14-07 | ARIB | AR IB |
| KT543_01-30-07 | ARIB | ARIB ARIIA |
| KT1070_02-22-07 | ARIB | ARIB, ARIIA, features of humoral rejection with transplant glomerulopathy |
| KT649_02-22-07 | ARIB | ARIB |
| KT673_02-14-07 | BL | Borderline |
| KT578_02-14-07 | BL | no definite AR, focal interstitial inflammation, potentially resolving AR |
| KT951_02-22-07 | BL | AR Borderline |
| KT559_02-12-07 | BL | borderline AR; CNIT |
| KT614_02-06-07 | STA | Normal |
| KT1239_2_18_09 | STA | Normal |
| KT641_06-25-07 | STA | Normal |
| KT387_02-07-07 | STA | Normal |
| KT901_06-28-07 | STA | Normal |
| KT905_06-25-07 | STA | Normal |
| KT417_02-07-07 | STA | Normal |
| KT588_02-06-07 | STA | Normal |
| KT889_06-28-07 | STA | Normal |
| KT903_2_16_09 | STA | Normal |
| KT839_06-25-07 | STA | Normal |
| KT860_2_16_09 | STA | Normal |
| KT929_2_16_09 | STA | Normal |
| KT977_06-28-07 | STA | Normal |
| KT540_02-06-07 | STA | Normal |
| KT612_02-06-07 | STA | Normal |
| KT922_01-26-07 | D0 | day zero |
| KT613_01-26-07 | D0 | day zero |
| KT616_06-25-07 | D0 | day zero |
| KT623_2_16_09 | D0 | day zero |
| KT636_01-24-07 | D0 | day zero |
| KT650_02-06-07 | D0 | day zero |
| KT651_02-14-07 | D0 | day zero |
| KT690-2_03-20-07 | D0 | day zero |
| KT707-1_2_16_09 | D0 | day zero |
| KT717_2_16_09 | D0 | day zero |
| KT730_01-26-07 | D0 | day zero |
| KT736_01-26-07 | D0 | day zero |
| KT738_06-25-07 | D0 | day zero |
| KT752_06-25-07 | D0 | day zero |
| KT755_02-06-07 | D0 | day zero |
| KT764_2_16_09 | D0 | day zero |
| KT1018 B_2_24_09 | D0 | day zero |
| KT776_05-25-07 | D0 | day zero |
| KT778_2_16_09 | D0 | day zero |
| KT781_02-01-07 | D0 | day zero |
| KT783-1_2_16_09 | D0 | day zero |
| KT796_05-25-07 | D0 | day zero |
| KT801_06-25-07 | D0 | day zero |
| KT812_05-25-07 | D0 | day zero |
| KT814_05-25-07 | D0 | day zero |
| KT816_06-25-07 | D0 | day zero |
| KT818_05-25-07 | D0 | day zero |
| KT819_06-25-07 | D0 | day zero |
| KT1422_1_26_11 | D0 | day zero |
| KT1441_1_26_11 | D0 | day zero |
| KT412_02-01-07 | D0 | day zero |
| KT575 | D0 | day zero |
| KT609_2_16_09 | D0 | day zero |
