# Supplementary material for: Significance and Suppression of Redundant IL17 Responses in Acute Allograft Rejection by Bioinformatics Based Drug Repositioning of Fenofibrate
Source: PLoS One. 2013 Feb 20;8(2):e56657. doi: 10.1371/journal.pone.0056657 (PMC3577752; doi:10.1371/journal.pone.0056657)
Supplement: Table S2 — Gene-sets of innate and adaptive immune cells (AcIc). (DOCX) [file pone.0056657.s002.docx]

**Table S2**: Gene-sets of innate and adaptive immune cells (AcIc)

| **Gene-Set** | **Genes** | **Affymetrix Probe-sets** | **Description** | **Ref.** |
| --- | --- | --- | --- | --- |
| Th1 | 260 | 408 | upregulated genes in in-vitro polarized Th1 cells compared to Th2 (n=5 each) (Affymetrix Hu6800: n=157 ProbeIDs, n=145 annotated Entrez_IDs; fc>2, p<0.05; ); pathway data of Th1 differentiation (n= 70 genes; SABioscience) | *[*[*1*](#_ENREF_1)*]* |
| Th2 | 133 | 242 | upregulated genes in in-vitro polarized Th2 cells and compared to Th1 cells (n=5 each) (Affymetrix Hu6800: n=58 ProbeIDs, n=52 annotated Entrez_IDs; fc>2, p<0.05; ); pathway data of Th2 differentiation (n= 81 genes; SABioscience) | *[*[*1*](#_ENREF_1)*]* |
| Th17 | 192 | 323 | upregulated genes in in-vitro activated Th17 cells vs. non-activated Th17 cells (n=3 each), (n=140 genes, fc 1.5; GSE11553; ABI Human Genome Survey Microarray v2.0); pathway data of Th17 differentiation (n=52 genes, SABiosciences) | *[*[*2*](#_ENREF_2)*]* |
| Treg | 192 | 2400 | upregulated genes in activated Treg vs. non-activated (fc>2) and not regulated in Th-cells (mRNAseq data; Illumina GAII) | *[*[*3*](#_ENREF_3)*]* |
| γδ-Tcells | 1078 | 1895 | upregulated genes (SAM, FDR 5%) in-vitro stimulated γΔTcells (vs. non-stimulated; n=3 each)(E-MEXP-1601, Affy U133plus2.0) | *Array Express: E-MEXP-1601* |
| NK | 16 | 27 | upregulated genes in IL15/IL12 stimulated NK cells (n=11) vs. non-stimulated (n=2) | *[*[*4*](#_ENREF_4)*]* |
| DC | 74 | 82 | upregulated genes in LPS activated vs. resting cells (n=6 each) | *[*[*4*](#_ENREF_4)*]* |
| Monocytes | 58 | 140 | freshly isolated cells (n=12) | *[*[*4*](#_ENREF_4)*]* |
| Neutrophils | 23 | 55 | freshly isolated cells (n=5) | *[*[*4*](#_ENREF_4)*]* |

**References**

1. Nagai S, Hashimoto S, Yamashita T, Toyoda N, Satoh T, et al. (2001) Comprehensive gene expression profile of human activated T(h)1- and T(h)2-polarized cells. Int Immunol 13: 367-376.

2. Cosmi L, De Palma R, Santarlasci V, Maggi L, Capone M, et al. (2008) Human interleukin 17-producing cells originate from a CD161+CD4+ T cell precursor. J Exp Med 205: 1903-1916.

3. Birzele F, Fauti T, Stahl H, Lenter MC, Simon E, et al. (2011) Next-generation insights into regulatory T cells: expression profiling and FoxP3 occupancy in Human. Nucleic acids research.

4. Abbas AR, Baldwin D, Ma Y, Ouyang W, Gurney A, et al. (2005) Immune response in silico (IRIS): immune-specific genes identified from a compendium of microarray expression data. Genes and immunity 6: 319-331.
