# Supplementary material for: Significance and Suppression of Redundant IL17 Responses in Acute Allograft Rejection by Bioinformatics Based Drug Repositioning of Fenofibrate
Source: PLoS One. 2013 Feb 20;8(2):e56657. doi: 10.1371/journal.pone.0056657 (PMC3577752; doi:10.1371/journal.pone.0056657)
Supplement: Materials and Methods S1 — Supporting materials and methods. (DOCX) [file pone.0056657.s003.docx]

**SM:** Material & Methods

**Fenofibrate**

Fenofibrate were purchased from Sigma Aldrich (F6020; Sigma Aldrich, St. Louis, MO). For in-vitro assays Fenofibrate was dissolved in DMSO 0.3% and supplemented with complete RPMI1640. For in vivo experiments, Fenofibrate was either suspended in sterile NaCl 0.9% (1078-20, Hospira Inc., Lake Forest, CA) and complemented with 0.5% Tween 20 (P9416; Sigma Aldrich, St.Louis, MO) and 0.5% Carboxymethylcellulose (419273; Sigma Aldrich, St.Louis, MO) at concentrations of 22.5mg/mL for daily oral gavage treatment of mice, or Fenofibrate was dissolved in sterile NaCl 0.9% complemented with Solutol HS15 USP grade, 15% (51633963, BASF, Germany) at a concentration of 7.5mg/mL for intra peritoneal (i.p.) treatment.

**IDs of gene expression assays used for QPCR of RNA from human PBMC**

IFNG (Hs00989291_m1); IL17A (Hs00174383_m1); 18S (Hs03003631_g1)

**IDs of gene expression assays used for QPCR of RNA from mice**

TNF (Mm00443258_m1); IFNG (Mm01168134_m1); STAT4 (Mm00448890_m1); IL6 (Mm00446191_m1); IL17A (Mm00439618_m1); TGFB1 (Mm01178820_m1); Jun (Mm00495062_s1); IL1B (Mm01336189_m1); CD127 (Mm00434295_m1); IL12B (Mm00434174_m1); 18S (Hs99999901_s1); STAT3 (Mm01219775_m1); RORA (Mm00443103_m1); RORC (Mm01261022_m1)

## RNA Extraction, Quality Control, Amplification, Hybridization

## For each kidney allograft biopsy, a separate biopsy core was placed in RNAlater (Ambion, Austin, TX) and stored at -20°C until RNA extraction. Total RNA was extracted from each biopsy using TRIzol Reagent (Invitrogen, Carlsbad, CA). RNA integrity was ensured using the RNA 6000 Nano LabChip Kit (Agilent Technologies, Waldbronn, Germany) on a 2100 Bioanalyzer (Agilent Technologies). RNA was amplified to cDNA and biotin labeled using the Ovation Biotin System (NuGEN Technologies, San Carlos, CA). The cDNA fragments were hybridized onto Affymetrix GeneChip Human Genome U133 Plus 2.0 Arrays comprising more than 54,000 probe sets, covering more than 47,000 transcripts and variants, including 38,500 well characterized human genes (Affymetrix, Santa Clara, CA). The microarrays were scanned using GeneChip Scanner 3000 (Affymetrix).

## In vitro experiments

Human PBMC were divided into three groups: non-stimulated negative control (NS) stimulated non-treated (S) and stimulated Fenofibrate treated (FF), each consisting of PBMC from the same 5 independent individuals and analyzed in triplicates. A total of 1.5x105 cells per well were plated on a 98 well plate (U-Bottom, Nunc, Roskilde, DK) in a total volume of 280µL RPMI 1640 supplemented with 10% FCS, Pen/Strep (100U/mL) and 2% non-essential Amino acids (all Gibco®, Invitrogen, Life Technologies, CA, USA). Prior to stimulation of cells, the FF group was treated with 100µmol Fenofibrate in DMSO 0.3% for 2 hours at 37°C and 5% CO2. Both groups FF and S were then stimulated with 8µL anti-Biotin MACSi ™ Bead Particles (2.5x107 loaded beads/ mL) bound to human CD3/-CD28-Biotin (1µg antigen /108 beads). Binding of antigens was performed according to the manufacture’s protocol (MACS human T-cell activation/ expansion kit, Miltenyi Biotec) for 2 hours at 4°C on a rotator. An equal volume of complete RPMI1640 was added to the unstimulated cells (NS). After 65 hours cells were centrifuged, washed with ice cold PBS (Gibco®, Invitrogen, Life Technologies, CA, USA), and frozen at -80°C until downstream analysis.

**Animals and heterotopic heart transplantation**

All animal protocols were approved by the Administrative Panel on Laboratory Animal Care at Stanford University (http://labanimals.stanford.edu/) and followed the NIH and USDA Guidelines for the Care and Use of Animals in Research [[1](#_ENREF_1)]. C57BL/6J (H2^b^) and FVB (H2^q^) mice were purchased from Jackson Laboratory (Bar Harbor, ME). Male mice (6 - 10 wk) with an average body weight of 25g were used in the experiments. Animals were maintained in the animal care facility at Stanford University. FVB donor hearts were implanted into the abdomen of C57BL/6 WT mice representing a complete MHC mismatch, as described previously [[2](#_ENREF_2),[3](#_ENREF_3)]. Briefly, through a midline abdominal incision, the aorta of the donor heart was anastomized to the recipient intrarenal abdominal aorta, and the pulmonary artery of the donor heart was anastomized to the inferior vena cava with 10-0 nylon suture. The donor ischemia time was approximately 45 min. Animals were divided into two treatment groups (Fenofibrate, Cyclosporine) and one non-treated control group for the POD30 graft survival model and into one treatment (Fenofibrate) and one non-treated control group for the POD7 Inflammatory model, each consisting of 6 animals. Animal activity, body weight and graft viability (abdominal palpation) were assessed daily. Mice were sacrificed with an inhalation overdose of isoflurane.

**Histology**

One third of the explanted allograft heart (POD7) was immediately fixed in 20% buffered formalin, embedded in paraffin and subsequently stained with hematoxylin and eosin for histological assessment of tissue according to standard protocols. Pictures of the graft tissue were taken at 10x magnification using a Nikon E600 light microscope (Nikon Instruments Inc., Melville, NY) and Spot V4.6 imaging software (Spot Imaging, Sterling Heights, MI).

**QPCR of RNA from mouse allograft and spleen tissue**

A total of 1.56ng of cDNA generated using Superscript II (Invitrogen Technologies Inc., Carlsbad, CA) was amplified in a target specific amplification step for 10 genes of interest in each sample using TaqMan PreAmp Master Mix (Life Technologies, Applied Biosystems, Foster City, CA) in the Eppendorf vapo.protect™ thermal cycler (Eppendorf, Hauppauge, NY) for a total of 18 cycles.

For QPCR preamplified cDNA was mixed with TaqMan Universal PCR Master Mix (Applied Biosystems) and Sample Loading Reagent (Fluidigm, San Francisco, CA) and pipetted into the sample inlets of a Dynamic Array 96.96 chip (Fluidigm). TaqMan gene expression assays (Applied Biosystems) for the 10 genes plus 18S as endogenous control gene were diluted with Assay Loading Reagent (1:2) (Fluidigm) and pipetted into the assay inlets of the same Dynamic Array 96.96 chip. After distributing assays and samples into the reaction wells of the chip in the NanoFlex controller (Fluidigm), the qRT-PCR reactions were performed in the BioMark RT PCR system for a total of 40 cycles. Data was analyzed using the BioMark RT-PCR Analysis Software Version 2.0.

Using the delta delta Ct method, gene expression in each sample was calculated relative to the expression in a universal RNA sample (human (740000) /mouse (750600) universal RNA, Stratagene, CA) in Microsoft Excel (Microsoft Office 2007, Microsoft Inc. USA).

## Flow cytometry of infiltrating cells in grafts at POD7

Single cell suspensions from recipients’ cardiac allografts at POD7 were prepared as follows: tissue was homogenized in RPMI 1640 media with 2 mg/mL collagenase D (Worthington Bio) and 10% FCS for 2 h at room temperature, cells were collected, washed twice in RPMI + 10% FCS, and counted after lysis of erythrocytes. Cells were then stimulated with 0.1**μ**g/ml PMA (Sigma-Aldrich) and 1 **μ**g/ml ionomycin (Sigma-Aldrich), and BrefeldinA (10**μ**L/6mL; BD Biosciences) for 4h at 37°C. After FcR block and addition f 5**μ**g/mL anti-CD16/32 mAb (2.4G2; BD Bioscience)), cells from cardiac allografts were stained with anti-mouse CD4 (GK1.5), CD8a (53-6.7), CD11b (M1/70), F4/80 (BM8), B220 (RA3-6B2), Gr1 (RB6-8C5), CD11c (N418), NK1.1 (PK136), CD45 (30-F11) mAb for 30 minutes at 4°C. Subsequently, 7-amino-actinomycin D (BD Bioscience) was added and incubated for 10 minutes. Fluorescein isothiocyanate (FITC), phycoerythin (PE), or allophycocyanin-conjugated mAbs specific for mouse CD4 (GK1.5), CD8a (53-6.7), CD11b (M1/70), F4/80 (BM8), B220 (RA3-6B2), Gr1 (RB6-8C5), CD11c (N418), NK1.1(PK136), CD45 (30-F11) , and their iso-type controls were purchased from BD Biosciences (San Jose, CA), eBioscience (San Diego, CA), or BioLegend (San Diego, CA). Expression of markers was determined by FACS Calibur (BD Bioscience) and FlowJo software (Tree Star).

**References**

1. National Research Council (1996) Guide for the Care and Use of Laboratory Animals. Washington, DC: National Academy Press.

2. Corry R, Russell P (1973) New possibilities for organ allografting in the mouse. Immunological aspects of transplantation surger. New York: Wiley. pp. 279.

3. Fischbein MP, Ardehali A, Yun J, Schoenberger S, Laks H, et al. (2000) CD40 signaling replaces CD4+ lymphocytes and its blocking prevents chronic rejection of heart transplants. J Immunol 165: 7316-7322.
